# Processing of abstract regularities in the human brain during dichotic listening

**DOI:** 10.1038/s41598-026-47637-w

**Published:** 2026-04-09

**Authors:** Petri Paavilainen, Leevi Karjalainen, Anni Nousiainen, Alma Nurmelin, Viivi Ovaskainen, Mari Tervaniemi

**Affiliations:** 1 Cognitive Brain Research Unit, Department of Psychology, University of Helsinki, (Haartmaninkatu 3), P.O.B. Box 21, Helsinki, FI-00014 Finland; 2 Department of Psychology, University of Helsinki, (Haartmaninkatu 3), P.O.B. Box 21, Helsinki, FI-00014 Finland; 3 Centre of Excellence in Music, Mind, Body, and Brain, Faculty of Educational Sciences, University of Helsinki, (Siltavuorenpenger 5), P.O.B. 9, Helsinki, FI-00014 Finland

**Keywords:** Mismatch negativity (MMN), Event-related potentials (ERPs), Attention, Memory, Auditory information processing, Neuroscience, Psychology, Psychology

## Abstract

The mismatch-negativity (MMN) component reflects automatic brain mechanisms encoding regularities in the auditory environment and detecting violations in them. Tone series, either descending or ascending in pitch, were used as stimuli. Occasional deviant stimuli (e.g., an ascending tone interspersed among descending tones) violated these regularities. The participants were presented either with a single descending binaural tone stream (identical input to both ears) or simultaneously with two different streams, an ascending stream to the left ear and a descending stream to the right ear. The effects of attention on the MMN were investigated by instructing the participants either to ignore the auditory stimuli or to detect the deviants in the designated ear. In the ignore conditions, the deviants in both the one-stream and two-stream conditions elicited the MMN. However, during the detection task, only the deviants presented in the attended ear elicited the MMN. The results demonstrate that the brain can encode opposite regularities from two simultaneously presented stimulus streams even when the participant’s attention is directed to another modality. However, the brain processes are not completely attention independent, as demonstrated by the lack of MMN to unattended-ear deviants when attention was firmly focused within the auditory modality to the other ear.

## Introduction

Patterns and rules embedded in the environmental stimulation in all modalities make it possible to predict features of the incoming stimuli based on the preceding stimuli. Predictability, in turn, may enhance information processing by allowing to direct limited cognitive resources to the processing of novel, potentially important stimuli, while stimuli having lower information value can be more or less ignored. In 1994, Tervaniemi and her colleagues presented their participants with tone sequences consisting of a repeating sequence of 12 steadily descending tones, presented with a 250-ms inter-stimulus interval (ISI) (e.g., ABCDEFGHIJKLABCDEFG.)^1^. Thus, the stimuli followed the rule that each tone was lower in frequency than the previous one (excluding, of course, the first tone of the repeating sequence). In one of their conditions, the stimulus sequences included occasional ascending tones that violated the rule. After their occurrence, the descending sequence continued (e.g., ABCDEFGH**G**HIJ.). Although the participants were ignoring the auditory stimuli and focusing on reading a book, these “direction deviants” elicited the mismatch negativity (MMN) component of the event-related potential (ERP). The occurrence of the MMN indicates that the brain has automatically (independently of attention) detected deviant events in the auditory environment. MMN has proven to be valuable both in the basic research on auditory information processing (for reviews, see^2–4^ and may also have potential clinical applications in determining dysfunctions related to various brain disorders^3,5^.

MMN is elicited when certain regular or invariant features of auditory stimulation are violated. Most MMN studies have employed an “oddball paradigm” where physically identical auditory stimuli (“standards”) are presented at short time intervals to the participant. The standards are occasionally replaced by “deviant” stimuli, such as tones of a different intensity, duration, or pitch. The MMN is seen in the ERPs to the deviant stimuli as a negative enhancement over the frontal and central scalp areas, reaching its maximum amplitude usually 150–250 ms after the onset of the deviant stimulus. In typical MMN experiments, the participant’s attention is deliberately directed elsewhere from the auditory modality (e.g., the participant is reading a book or attending to a video during the auditory stimulation). In such an ignore condition, MMN is not obscured by overlapping ERP components related to controlled processing mechanisms, such as the N2b^6,7^. In attend conditions, where the participant’s task is to detect the deviant stimuli, the N2b is additionally followed by the large parietal P3b component, reflecting conscious categorisation and working memory operations^8^.

As a theoretical explanation for the MMN elicitation, Risto Näätänen and his colleagues originally proposed that the physical features of the repeating standard stimulus are encoded in rapidly decaying memory traces at the auditory cortices (e.g.,^9,10^). The MMN reflects a mismatch between the new auditory input and the contents of the memory trace. The above-described paradigm used by Tervaniemi et al. was, however, specifically noteworthy as there was no physically constant, repeating standard stimulus at all. Instead, each standard tone was physically different from the previous tone (having a lower pitch). In contrast, the deviant stimuli (ascending tones) involved a physically similar stimulus that had just occurred in the immediate past. This kind of MMN has been termed abstract-feature MMN as its elicitation seems to imply that the brain had managed to extract a more complex, “abstract” invariance from the physically varying stimuli, such as the descending tone pattern (for a review, see^11^.

Another abstract MMN paradigm had been earlier developed by Saarinen et al.^12^. They presented their participants with tone pairs, consisting of two 60-ms tones with a 40-ms inter-tone gap. However, the pitches of the tones varied randomly. Consequently, there was no identically repeating standard stimulus but rather a class of physically different standard stimuli, sharing a common “higher-order” feature: all the different exemplars of the standard pairs were ascending, i.e., the second tone of the pair was always higher in pitch than the first tone. The deviant pairs, in turn, were descending in their within-pair pitch direction. The deviant pairs elicited the MMN, which was interpreted as suggesting that the proposed memory traces were able to derive certain “abstract” invariances (“rise”, “fall”) from auditory stimulation varying on its physical “surface” features.

According to a theory proposed by Winkler, the brain mechanisms generating MMN automatically extract various regularities from the auditory environment and encode them in neural models^13^. If the regularities change, the models are updated. The MMN signals the updating process that follows if stimuli violating the encoded regularities are received. Additionally, the MMN generator mechanism has been proposed to initiate an involuntary attention switch to abrupt changes in the auditory environment, as they may carry potentially important information. This redirection of attentional resources would ensure the adequate processing of auditory stimuli even in situations where attention was initially directed, for example, to visual modality^14,15^.

However, the extent to which MMN generation actually is independent of attention has been a controversial issue during the past decades (for a review, see^16^. The typical primary tasks used in the ignore conditions (such as video watching) do not allow for effective control over the direction of the participants’ attention. Their attention may occasionally “leak” also into the auditory modality. Using attentionally more demanding primary tasks, it has been shown that the direction of attention may indeed modulate the MMN amplitude. In the 1990s, several MMN experiments were conducted using dichotic listening tasks to better control the direction of the participants’ attention. Two separate streams of oddball tones were delivered, one stream to the participant’s left ear and another to the right ear (e.g.,^17–20^). The participants’ task was to attend to the tones presented, e.g., in the right ear, and to press a reaction key whenever they noticed a deviant in that ear.

Interpreting the results from the dichotic listening experiments was, however, somewhat ambiguous. In some studies, attenuation or even total suppression of MMN to unattended-ear deviants was observed. On the other hand, the MMN recorded to the attended-ear deviants often seemed to be larger in amplitude. However, this enhancement could be, at least partially, explained by the attention-related N2b component overlapping the MMN rather than being due to the more vigorous activation of the MMN generator mechanism per se. The magnitude of the attention effects also seemed to depend on the physical feature eliciting the MMN (i.e., frequency, intensity) and on the attribute used as the target in the attended ear. According to the so-called competition hypothesis^21^, selective attention does not enhance or attenuate the MMN generator proper. However, top-down processes may modulate the type of information reaching the deviance-detection process when changes in several simultaneous information channels are competing for the same resources.

However, the effects of attention on the processing of abstract deviants have not been extensively studied. Paavilainen et al.^22^ employed Saarinen et al.’ s^12^ tone-pair paradigm in a dichotic-listening experiment, where standard and deviant tone pairs were presented at a rapid rate to the participant’s left and right ears, the abstract attributes (ascending/descending) being opposite in the two ears. To increase the perceptual separability of the two tone streams, the left-ear pairs were presented on a lower pitch area than the right-ear pairs. In an ignore condition (participant reading a book), deviant pairs in both ears elicited MMN, indicating that the brain was able to extract, separately for the two ears, opposite abstract attributes from two rapid, concurrent stimulus streams. When attention was focused on detecting the left-ear deviants, MMN was still elicited by the unattended right-ear deviants. However, when attention was focused on the right ear, the unattended left-ear deviants did not elicit MMN. The authors speculated that abstract attributes in the left- and right-ear inputs might be processed differently. The auditory areas in the left hemisphere could be more specialised for that type of analytical processing required in extracting abstract attributes. As the processing of the right-ear input occurs predominantly in the left auditory cortices, it might be less vulnerable to the effects of intense focusing of attention to the opposite ear.

The present study has two main goals. First, we aimed to investigate further how complex, concurrent patterns the brain can extract from the auditory environment. Tervaniemi et al.‘s^1^ paradigm is applied to a dichotic listening experiment where two different tone streams are simultaneously presented: the left-ear tones are ascending in pitch, whereas the right-ear tones are descending. Both streams include deviant tones whose direction is opposite to the rule in that ear. To further enhance the separability of the left and right-ear inputs, the descending right-ear sequences are presented on a lower pitch area than the ascending right-ear sequences (a similar pitch cue was also used by Paavilainen et al.^22^ in their dichotic-listening experiment). For comparison and to confirm the replicability of the original Tervaniemi et al. MMN effect^1^, also binaural stimulus blocks are included, where a single, descending tone stream is presented to both ears. These blocks are in stimulus parameters identical to the Tervaniemi et al. study (their condition with sinusoidal tones and ascending deviants). Also, the temporal dimensions of the original study are kept similar in terms of sound duration and presentation rate.

The second goal is to study the possible attention effects on the processing of abstract direction deviants. The study consists of both ignore and attend conditions. In the ignore conditions, the participants will be watching a video during the auditory stimulation. Of special interest is the dichotic ignore condition: will the direction deviants both in the left-ear and in the right-ear inputs elicit the MMN? In the attend conditions, a more stringent control of attention is used. The participants will direct their attention either to the left-ear or right-ear input, and their task is to detect direction deviants in that ear. Will the direction deviants in the opposite, unattended ear still elicit the MMN?

Consequently, the main interest in the present study is in the ignore and unattended-ear data. Our first hypothesis is that in the binaural stimulus blocks, the original Tervaniemi et al.^1^ MMN effect will be replicated. For the dichotic stimulus blocks with two opposite stimulus streams, we hypothesize that in the ignore condition, the deviant tones in both streams will elicit MMN. This would conceptually replicate the ignore-condition results of Paavilainen et al.^22^ The unattended-ear MMN results in the attend conditions of Paavilainen et al. were not consistent between the left and right ears (as described above). Consequently, we did not specify in advance any hypothesis for the present unattend-ear data regarding whether MMN should or should not appear there. For the sake of comparison, we will also present the attended-ear data, although it should be kept in mind that the present study was not designed to allow the separation of MMN from the attention-related N2b component.

## Methods

### Participants

Sixteen adults (12 females, four males; age range 20–22 years; all right-handed) participated in the experiment. The number of participants was set before the experiments. However, as one participant’s data was lost due to a technical problem, an additional participant was obtained to replace the missing data. All the participants reported having normal hearing. They received course credits for their participation in the study. Informed written consent was obtained from all participants. The experimental procedure was carried out in accordance with the Declaration of Helsinki, and it was ethically accepted by the University of Helsinki Ethical Review Board in Humanities and Social and Behavioural Sciences (approval number: 2/2025).

### Stimuli and procedure

**Fig. 1:**
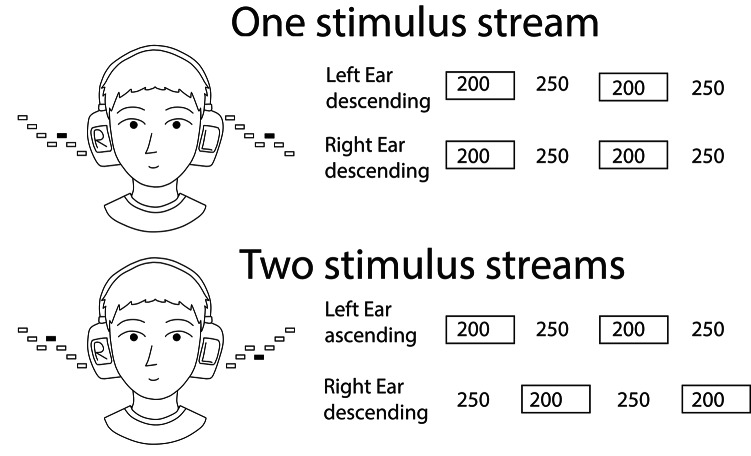
A schematic illustration of the experimental paradigm and the timing of the auditory stimulation (numerical values are in ms). In the one stimulus stream blocks (upper panel), the same descending tone stream was presented simultaneously to both ears (the black rectangles in the figure of the participant’s head denote the deviant stimuli). In the two stimulus stream blocks (lower panel), a descending tone stream was presented in the left ear and an ascending tone stream in the right ear. Left and right ear tones were presented in an alternating order so that left-ear tones were presented during the silent right-ear interstimulus intervals and vice versa. Illustration created by Andreea Carmen Dumitrescu and Petri Paavilainen.

Two types of stimulus blocks were used (see Fig. 1). In the *one-stream* blocks (in the following, 1-STR), the same repeating, descending sequence of 12 sinusoidal tones was presented binaurally to both ears via earphones. The tones (duration 200 ms with 10-ms rise and fall times; intensity about 70 dB) were delivered with an interstimulus interval (ISI) of 250 ms. The frequencies of the tones varied between 311 Hz (corresponding to D4# on a musical scale) and 587 Hz (D5) in semitone steps. The tones were presented in circularly looped sequences in a descending order (587 Hz, 554 Hz, 523 Hz,…311 Hz). The next 12-tone sequence started with the 250-ms ISI immediately after the previous sequence was finished. The stimulus sequences included as deviant events (*p* = 0.10) occasional tones *ascending* to the frequency of the preceding tone, after which the descending sequence continued (e.g., …554 Hz, 523 Hz, 494 Hz, **523 Hz**, 494 Hz…). The stimulus parameters described above were identical to those in the original Tervaniemi et al. study^1^(their condition with sinusoidal stimuli and ascending deviants). The earliest position in the 12-tone sequence where a deviant could appear was the 3rd position. The binaural blocks consisted of 1000 tones. The duration of each block was 7.5 min.

In the *two-stream* blocks (2-STR), a low, descending stimulus sequence was presented to the right ear and a high, ascending stimulus sequence to the left ear. The parameters of the right-ear sequence were identical to those in the binaural stimulus blocks. The frequencies of the left-ear stimuli were higher than those of the right ear: they varied with semitone steps between 1245 Hz (D6#) and 2349 Hz (D7). They were presented in an ascending order (1245 Hz, 1319 Hz, 1397 Hz,…2349 Hz). The right-ear sequences included occasional ascending tones (*p* = 0.10) and the left-ear sequences occasional descending tones (*p*=0.10) as deviant events. In both sequences, the earliest position where a deviant could appear was the 3rd position. Tones were presented in an alternating order to the left and right ear so that the left-ear tones were presented in the middle of the silent right-ear inter-stimulus intervals and vice versa (see Fig. 1). Thus, the temporal parameters of the left-ear and right-ear stimulus streams were similar to those in the 1-STR blocks. The 2-STR blocks consisted of 2000 tones (i.e., 1000 left-ear and 1000 right-ear tones). The duration of each block was 7.5 min.

Both the 1-STR and 2-STR blocks were presented in two conditions. In the *ignore conditions*, the participants were watching a silent video with subtitles (Walt Disney’s *Pinocchio*) while the auditory stimuli were presented. The participants were instructed to concentrate on watching the video, and they were not required to pay any attention to the auditory stimuli. In the *detection conditions*, the video was paused. The participants were instructed to concentrate on listening to the auditory stimuli and to press a button with their right-hand index finger always when they noticed a designated deviant tone, serving as a target. In the 1-STR blocks, the target tones were the ascending deviants. In the 2-STR blocks, there were two types of attend conditions. In the *attend left condition*, the participants’ task was to concentrate on listening to the left-ear tones and to press the button to the descending deviants presented in that ear. In the *attend right condition*, the participants’ task was to concentrate on listening to the right-ear tones and to press the button to the ascending deviants presented in that ear.

The 1-STR and 2-STR blocks were presented in a randomised order. The ignore and attend conditions were presented in an alternating order, with half of the participants starting with an ignore condition and half with an attend condition. In the attend conditions, each stimulus block was presented once, and in the ignore conditions twice (in order to prevent the experiment from becoming too long and stressful for the participants, the cognitively more demanding attend conditions were presented only once). Thus, the experiment consisted of 7 stimulus blocks (two 1-STR ignore, one 1-STR attend, two 2-STR ignore, one 2-STR attend left, one 2-STR attend right). With preparations, the experiment lasted about two hours.

During the experiment, the participants sat in a comfortable reclining chair in an electrically and acoustically shielded room. A computer terminal (diameter 63 cm) for watching the video was placed about 130 cm in front of the participant. The participants were instructed to avoid excessive blinking and body movements during the EEG measurements.

### ERP recording and statistical analysis

The EEG (sampling rate 250 Hz, bandpass 1–30 Hz; -6 dB cutoff frequencies 0.5 Hz and 33.75 Hz, respectively) was recorded with Ag/AgCl electrodes placed at Fpz, Fz, F3, F4, Cz, Pz, and the left (LM) and right (RM) mastoids. The vertical eye movements were recorded with an electrode above the left eye, and the horizontal eye movements with an electrode placed at the outer canthus of the left ear. The reference electrode was attached to the tip of the nose, and the grounding electrode was on the forehead.

The EEG was cut to 500-ms epochs starting 100 ms before the onset of the tone. The epochs containing EEG or EOG changes over ±70 µV were omitted from the averaging. The various exemplars of the standard tones (obeying the descending/ascending rule) in the different positions of the 12-tone sequences were averaged together. The same was done for the different exemplars of the direction deviants. The grand-average ERPs were calculated by averaging together the corresponding ERPs from the 16 participants. Difference waves were calculated by subtracting the ERPs to the standard stimuli from those to the deviants. The 100-ms period preceding the onset of the stimulus was used as a baseline in the ERPs.

The MMN amplitudes were calculated from the difference waves as the mean amplitude at Fz during 150–250 ms. Based on the previous MMN studies, these were selected as representing the electrode and latency zone, where the MMN is typically most prominent. The presence of MMN in the various conditions was determined with t-tests by comparing the MMN amplitudes to zero. The normality assumptions for using t-tests were generally met as indicated by the Shapiro-Wilk tests (see, however, Table 1). One-tailed tests were used as the difference was predicted to be negative. One-way ANOVA was used to study the differences in MMN amplitudes between the conditions, as Levene’s test indicated no evidence of unequal variances.

The hit rates (percentage of the target deviants detected) and mean reaction times (RTs) for the detected target deviants were calculated. A response was classified as a hit if the button press occurred between 200 and 900 ms from the onset of the target deviant.

## Results

**Fig. 2:**
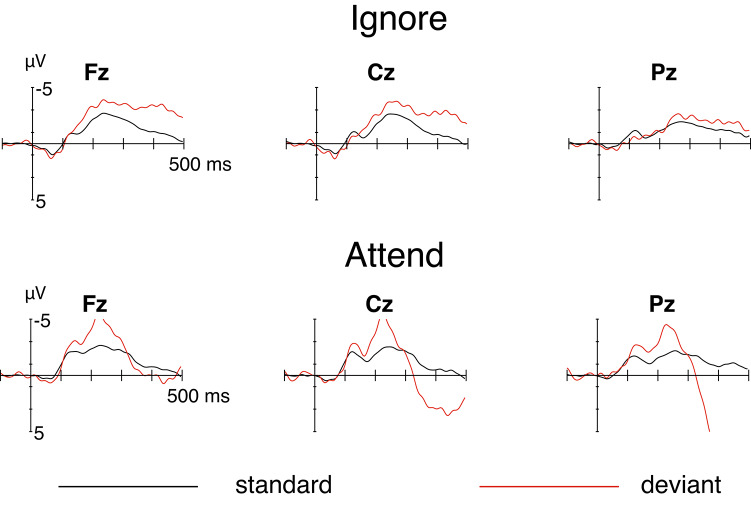
The ERPs at midline electrodes Fz, Cz, and Pz to standard (black) and deviant (red) stimuli in the one stream condition (upper row: ignore; lower row: attend). The onset of the stimulus is at the intersection of the x and y axes.

The ERPs in the 1-STR blocks are presented in Fig. 2, separately for the ignore (top row) and attend (bottom row) conditions. In the ignore condition, the MMN is seen in the ERPs to deviants as a frontally distributed negative enhancement, starting at about 150 ms. This result replicates the MMN obtained by Tervaniemi et al.^1^.

Also in the attend condition, an MMN is seen in the deviant-stimulus ERPs, followed by a sharper, centrally distributed N2b component (peaking at about 230 ms) and a large, late parietal P3b component. The MMNs were statistically significant in both conditions (see Table 1), although in the attend condition, the amplitudes during the measurement window (150–250 ms) were obviously enhanced by the partially overlapping N2b. However, during the early part of the measurement window (150–200 ms), the deviant-stimulus ERPs were very similar in the ignore and attend conditions, suggesting that attention did not have any major enhancing effect on the MMN. The MMN mean amplitudes during 150–200 ms did not differ significantly between the ignore and attend conditions (t(15) = 0.09, *p* = 0.9307, Cohen’s d = 0.02).

**Table 1:** Fz mean amplitudes (µV) during 150–250 ms in the deviant minus standard difference waves in the various conditions, and the corresponding standard deviations, t-values, p-values, and effect sizes (Cohen’s d).

	1-stream binaural blocks		2-stream dichotic blocks					
			Left-ear stimuli			Right-ear stimuli		
Condition:	Ignore	Attend	Ignore	Attend left	Attend right	Ignore	Attend left	Attend right
Amplitude	−1.08	−1.76	−0.69	−1.38	−0.20	−0.75	0.12	−0.98
SD	1.01	2.03	1.15	1.45	1.55	1.23	1.27	1.56
t(15)	−4.27	−3.48	−2.41	−3.79	−0.52	−2.45	0.37	−2.50
p	0.0007	0.0017	0.0293	0.0009	0.3047	0.0271	0.6417	0.0122
Cohen’s d	−1.07	−0.87	−0.60	−0.95	−0.13	−0.61	0.09	−0.62

The mean amplitudes were compared to zero with one-tailed t-tests. The eight p-values are reported here without correction for multiple tests, but the statistically significant values remained significant even after the Benjamini-Hochberg False Discovery Rate correction. In the attend-right condition for the right-ear stimuli (rightmost column), the normality assumption for the t-test was not met in the Shapiro-Wilk normality test. However, the comparison differed statistically significantly from zero even when using a nonparametric one-sample Wilcoxon Signed Rank test (V = 28; *p* = 0.0366). The statistical power for detecting MMN effects was very good in the 1-STR ignore condition (0.99) and moderate in 2-STR ignore conditions (about 0.74).

**Fig. 3:**
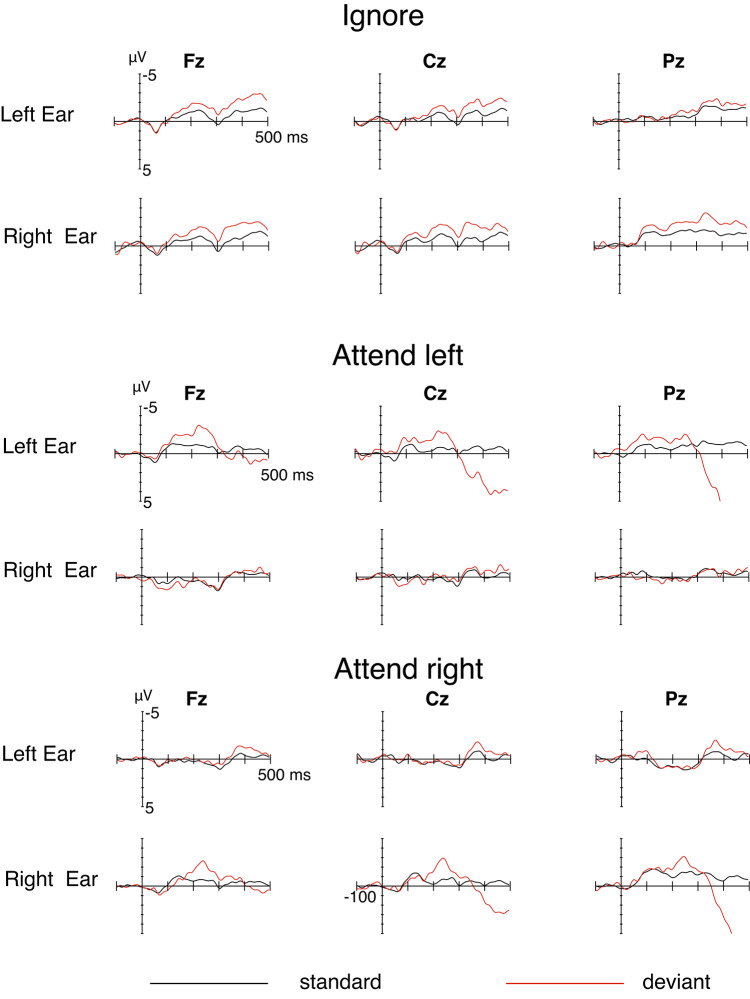
The ERPs at midline electrodes Fz, Cz, and Pz to standard (black) and deviant (red) stimuli in the two-stream conditions, separately for the left and right ear stimuli. Top panel: Ignore condition (video watching). Middle panel: Attend left-ear deviants. Bottom panel: Attend right-ear deviants. The onset of the stimulus is at the intersection of the x and y axes.

Figure 3 shows the ERPs obtained in the 2-STR blocks in the various conditions. In the ignore condition, the deviant stimuli both in the left and right-ear inputs, elicited the MMN (1st and 2nd rows in Fig. 3). Both MMNs were statistically significant (Table 1). However, in the attend-left condition, the unattended right-ear deviants did not elicit any MMN (4th row). A similar result was obtained in the attend-right condition: the unattended left-ear deviants did not elicit MMN (5th row). In turn, the attended-ear deviants in both ears elicited MMN, followed by large N2b and P3b components (3rd and 6th rows), the latter components indicating that the attention had indeed been focused on the designated ear and the participant had been detecting the deviants as instructed. Statistically significant MMNs were obtained in both ears. However, as the MMN amplitude is probably again enhanced by the partially overlapping N2b component, the present data do not allow us to determine whether directing the attention to the deviants enhances the MMN generator per se, compared to the ignore condition. Somewhat puzzlingly, in the attended right-ear data (6th row), there seems to be no early MMN during 100–150 ms when compared to the corresponding left-ear data (3rd row).

**Fig. 4:**
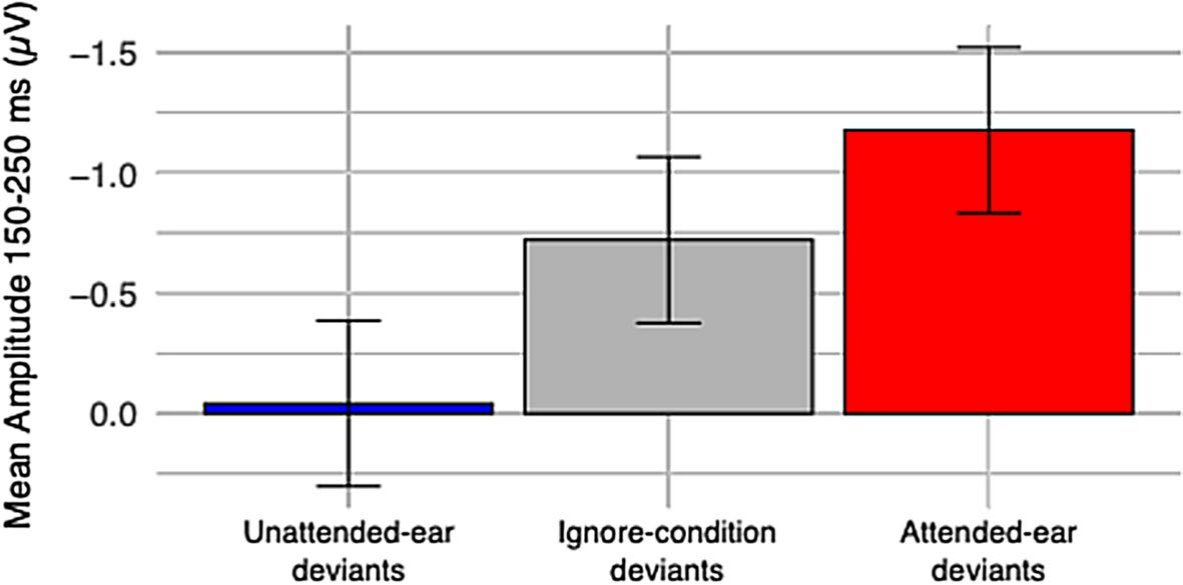
The Fz mean amplitudes (150–250 ms) measured from the deviant minus standard difference waves in the two-stream conditions for the unattended-ear deviants recorded during dichotic listening (blue), ignored deviants recorded during video watching (grey), and attended-ear deviants recorded during dichotic listening (red). The corresponding left and right ear data were combined. Error bars denote the standard error of the mean.

As the left-ear and right-ear data were very similar in all 2-STR conditions, they were combined for a 1-way ANOVA studying the effect of attention on the MMN amplitude (conditions: unattended ear, attended ear, ignore). The effect of attention was statistically significant (F(2,45) = 4.37, *p*<0.019, n^2^ = 0.16). Tukey’s post hoc test indicated that the unattended ear vs. attended ear comparison was statistically significant (*p*<0.014). The results are illustrated in Fig. 4. It is again important to note that the partially overlapping N2b component probably enhances the MMN amplitudes in the attended-ear data.

The mean reaction times in the 1-STR attend, 2-STR attend left, and 2-STR attend right conditions were 541 (SD = 51), 546 (61), and 556 (54) ms, respectively. The corresponding hit rates were 66 (21), 64 (23), and 58 (22) %. False alarm rates were low, 1.4, 1.4, and 1.8%.

## Discussion

The present study was designed to replicate and extend the findings of Tervaniemi et al.^1^ For comparison and to confirm the replicability of the original Tervaniemi et al. MMN effect, binaural blocks with a single stimulus stream were presented with parameters identical to those used in the original experiment. To extend the findings, their paradigm was applied in a dichotic listening experiment where two different tone streams were simultaneously presented, one to the left ear and another to the right ear. The regular patterns were opposite between the two ears, the left ear sequences being ascending and the right-ear sequences descending. Both streams included deviant tones whose direction was opposite to the rule in that ear.

First, in the 1-STR ignore condition, the result of Tervaniemi et al. was replicated, and a clear, statistically significant MMN was obtained. However, the most interesting finding of the present study was that also in the 2-STR ignore condition, MMN was elicited by the deviants presented both in the left and right ears. Thus, even when the participants’ attention was directed away from the auditory stimuli to another modality, the brain can extract opposite abstract attributes (ascending/descending) from two different auditory stimulus streams. However, instead of a sharp transient deflection, the present MMNs were more like long-lasting negative shifts. Possibly, the time-locking of the MMN to the onset of the deviant stimulus is less precise with abstract deviants, or the neural deviance detection process takes more time with such stimuli. The present results are in line with the results of Paavilainen et al.^22^. They obtained MMNs to abstract deviants in both ears in the ignore condition of their dichotic-listening study, using tone pairs as stimuli (descending in one ear and ascending in another). It should be noted, however, that the MMN amplitudes in the 2-STR ignore condition, both for the left and right-ear deviants, were somewhat smaller than the MMN amplitude in the 1-STR ignore condition (although the differences were not statistically significant). However, they may indicate that the extraction of two concurrent regularities does not occur totally parallel and independently of each other, but there may be some “cost” to be paid for the brain compared to the extraction of a single regularity.

To detect the deviants in both ears, the brain must somehow be able to separate the two concurrent stimulus streams from each other and extract the regularities specific to each stream. Similar to Paavilainen et al.^22^ study, the present study offered two physical cues for this purpose: the ear of input and the pitch level: The left-ear sequences were presented on a higher pitch level than the right-ear sequences. As it is known that 2/3 of the connections from each ear are directed to the contralateral auditory areas (see, e.g.,^23^), one possibility would be that the left auditory cortex was predominantly responsible for extracting and maintaining the regularity model for right-ear stimuli and *vice versa.* On the other hand, it is also possible that the pitch cue was responsible for separating the auditory stimuli into two separate streams. From the auditory streaming studies, it is known that with rapid stimulation pace, high and low pitch stimuli can be perceptually separated into two different streams^24^. This phenomenon has also been shown in MMN experiments utilising the auditory streaming effect (see, e.g.,^25^). It is, of course, possible that both the ear and pitch cues contributed to the present MMN effect. It should also be noted that as the two stimulus streams were independently randomized, occasionally two deviants, one in the right-ear stream and another in the left-ear stream, could follow each other with very close succession. In these cases, both deviants may elicit their own, temporally (partially) overlapping deviance detection processes. However, the current setup did not allow separation of their contributions in the ERPs.

The second goal was to study the possible attention effects on the processing of direction deviants. The 2-STR attend condition results demonstrated that the MMN mechanism was clearly influenced by attentional, top-down influences within the auditory modality: When attention was strongly focused on the left ear, the right-ear deviants no longer elicited MMN. The same phenomenon was observed for the left-ear deviants when the right ear was attended to. The fact that the attended deviants elicited prominent N2b and P3b components, while both components were completely missing from the ERPs to the unattended-ear deviants, confirms that the participants focused their attention successfully according to the instructions. Also, the behavioural measures indicate that the participants were concentrating on their detection task, which, however, obviously was somewhat demanding, as evidenced by the far from perfect hit rates. Previous dichotic-listening studies have shown that when the participant’s task is to detect targets in the attended ear based on a designated physical feature, the MMNs to deviations in the same physical feature are strongly reduced in the unattended ear (e.g.,^26^). This kind of competition for the same processing results^21^ could also explain the disappearance of the abstract MMN in the unattended ear in the present study, as in both ears the abstract feature was based on the same principle (ascending/descending tone sequence).

In their dichotic-listening study with abstract tone pairs, Paavilainen et al. observed in an attend-right condition MMN to the unattended left-ear deviants. However, no similar effect was found in the right-ear data in the attend-left condition. They speculated that hemispheric differences in processing abstract features could explain this asymmetry. However, a similar effect could not be found with the present stimuli: the MMN was missing in both the unattended left-ear and unattended right-ear data. The discrepancy between the present results and those of Paavilainen et al. might be due to different types of abstract regularities used (ascending/descending tone series vs. ascending/descending tone pairs). Another possibility is that the MMN to unattended left-ear stimuli found by Paavilainen et al.^22^ was just a false positive finding (or alternatively, the present absence of MMN was a false negative finding).

However, the visual inspection of the unattended ear ERPs (Fig. 3) reveals two effects that might be kept in mind for future studies. Both the standard and deviant ERPs in the unattended ear data seem to be suppressed compared to the ignore condition. Thus, strong focusing of attention to the opposite ear seems to have a general dampening influence on the processing of auditory input in the unattended ear (see, e.g.^17^). However, in the attend-right condition, this suppression seems to be released in the left-ear data after 300 ms, where even a small late negative difference between the deviant and standard ERPs is emerging. However, if this is a late MMN effect, it is not consistent with the results of Paavilainen et al., who observed their unattended-ear MMN effect only in the right ear.

The present results are more challenging to interpret regarding the opposite question: did intense focusing of attention on the auditory stimuli enhance the MMN to the attended deviants? As the attended deviants were also the targets, the MMN was succeeded and partially overlapped by the N2b component. The mean amplitudes during the 150–250 ms measurement window for the attended deviants were clearly larger compared to those in the ignore conditions (Table 1; Fig. 4). Still, this enhancement is obviously caused (at least predominantly) by the partially overlapping N2b component. One possibility to circumvent this interpretational problem in future studies might be to change the setup so that in the attend conditions, the direction deviants are not the target stimuli. Instead, the targets could be, e.g., occasional lower-intensity standard stimuli, similarly to those in a recent study by Paavilainen and Ilola^27^. This way, the target deviants would elicit the N2b, and while the direction deviants would still be in the attended input, they are expected to elicit no N2b. Another methodological recommendation for future studies would be to counterbalance the directions of the stimulus streams between the left and right ears, as now in the 2-STR conditions, the descending sequence was always presented to the right ear and the ascending sequence to the left ear. One reason for the puzzling absence of MMN in the attend-right 2-STR condition might have been the use of a rather high 1 Hz high-pass filter, which has been shown in ERPs with pronounced P3 components to flatten activity also in other latency ranges^28^. Consequently, in future studies, a less extreme high-pass filtering (e.g., 0.5 Hz) might be recommended. Finally, larger sample sizes are recommended to be used in similar future studies to increase the statistical power for detecting small MMN effects.

In conclusion, the major finding in the present study was the clear MMN obtained in the 2-STR ignore condition. The deviants in both the left and right ears elicited a statistically significant MMN, further increasing the credibility of the effect. This result shows the remarkable ability of the brain to extract opposite abstract regularities from two concurrent stimulus streams, even when the participant’s attention is directed away from the auditory modality. Equally apparent was the disappearance of MMN in the unattended-ear data when attention was firmly focused on the opposite ear, demonstrating that the MMN mechanism is not totally independent of within-modality attentional influences.

## Data Availability

The datasets generated during and/or analyzed during the current study are available from the corresponding author on reasonable request.
